# Nipecotic Acid Derivatives as Potent Agents against Neurodegeneration: A Preliminary Study

**DOI:** 10.3390/molecules27206984

**Published:** 2022-10-17

**Authors:** Georgios Papagiouvannis, Panagiotis Theodosis-Nobelos, Eleni A. Rekka

**Affiliations:** 1 Department of Pharmacy, School of Health Sciences, Frederick University, 1036 Nicosia, Cyprus; 2 Department of Pharmaceutical Chemistry, School of Pharmacy, Aristotle University of Thessaloniki, 54124 Thessaloniki, Greece

**Keywords:** Alzheimer’s Disease, multi-targeting compounds, nipecotic acid, oxidative stress, inflammation, acetylcholinesterase inhibitors

## Abstract

Alzheimer’s Disease (AD) is a common neurodegenerative disorder characterized by memory loss and cognitive impairment. Its pathology has not been fully clarified and therefore highly effective treatments have not been obtained yet. Almost all the current treatment options aim to alleviate only the symptoms and not to eliminate the disease itself. Acetylcholinesterase inhibitors are the main therapeutic agents against AD, whereas oxidative stress and inflammation have been found to be of great significance for the development and progression of neurodegeneration. In this work, ethyl nipecotate (ethyl-piperidine-3-carboxylate), a heterocyclic carboxylic acid derivative, which acts as a GABA reuptake inhibitor and has been used in research for diseases involving GABAergic neurotransmission dysfunction, was amidated with various carboxylic acids bearing antioxidant and/or anti-inflammatory properties (e.g., ferulic acid, sinapic acid, butylated hydroxycinnamic acid). Most of our compounds have significant antioxidant potency as lipid peroxidation inhibitors (IC_50_ as low as 20 μΜ), as oxidative protein glycation inhibitors (inhibition up to 57%), and act as DPPH reducing agents. Moreover, our compounds are moderate LOX inhibitors (up to 33% at 100 μΜ) and could reduce rat paw edema induced by carrageenan by up to 61%. Finally, some of them possessed inhibitory activity against acetylcholinesterase (IC_50_ as low as to 47 μΜ). Our results indicate that our compounds could have the potentiality for further optimization as multi-targeting agents directed against AD.

## 1. Introduction

Alzheimer’s Disease (AD) is the most prevalent neurodegenerative disorder, affecting millions of people all over the world [1]. It is an age-related disease, characterized by symptoms such as memory deficiency, cognitive impairment, and various behavioural symptoms [2]. Its main histopathological lesions include extracellular aggregates of Aβ amyloid peptide, deriving from amyloid precursor protein (APP) cleavage and intracellular neurofibrillary tangles (NFTs) from hyperphosphorylated tau protein aggregation [3]. Both amyloid deposits and NFTs induce neurotoxicity, neurodegeneration, and dysfunction of synapses [4]. Although significant progress in understanding AD pathophysiology has been made, the mechanisms that lead to initiation and development of AD have not been fully clarified. Therefore, most of the treatment options concern the symptomatic alleviation and not the elimination of the disease itself. Oxidative stress has been found to play a key role in neuronal degeneration. Toxicity of oxygen can lead to various dysfunctions in the Central Nervous System (CNS), since about 20% of the respirated oxygen is consumed by the brain [5]. Beta- and gamma-secretase function is induced by oxidative stress; therefore, amyloid peptide aggregation increases [6]. In addition, advanced glycation end-products (AGEs), formed due to extensive oxidative damage, increase amyloid precursor protein (APP) concentration and thus amyloid plaque formation, modulate tau protein phosphorylation, and induce gene expression leading to cell apoptosis [7]. Mitochondrial dysfunction, deriving from oxidative stress has been indicated as an underlying mechanism for AD pathology, and is highly associated with cell death, tau protein hyperphosphorylation, and neurodegeneration [8,9]. Finally, excitotoxicity caused by hyperactivity of N-methyl-D-aspartate (NMDA) receptors, leads to elevated Ca^2+^ levels, reactive oxygen species (ROS) production, and thus to cell dysfunction and death [10].

Inflammation is another mechanism involved in AD progression. Acute inflammation is associated with neuroprotection, whereas chronic inflammation causes neurotoxicity through cytokines release and ROS production [11]. Activation of microglial cells and astrocytes is responsible for functional and structural changes in brain cells, leading to apoptosis and neurodegeneration [12]. Prostaglandins, formed by arachidonic acid cleavage mediated by Cyclooxygenase (COX), are considered key factors in AD development [13]. Especially PGE2 has been found to inhibit phagocytosis of β-amyloid peptides and enhances neurotoxic activity of microglia [14]. Moreover, both amyloid plaques and NFT formation are highly associated with lipoxygenase (LOX) activity, which has been found to increase with age [15].

According to the cholinergic hypothesis, cholinergic neuron depletion, and the subsequent decrease in cholinergic neurotransmission, contribute to memory and cognitive impairment during AD [16]. Low concentrations of acetylcholine (Ach) have been observed in brains of patients with AD, and increase of the neurotransmitter levels through acetylcholinesterase (AchE) inhibition has proved useful for symptom amelioration [17]. Failure to treat AD effectively using AchE inhibitors has led to cholinergic hypothesis reconsideration and deeper study of the relations of AchE with other factors participating in AD pathogenesis, such as deposition of amyloid plaques and NFT formation [18,19,20,21].

Considering all the above-mentioned evidence, and following the multi-targeting molecules strategy [22], we propose that compounds with antioxidant and/or anti-inflammatory properties could enhance the effort for obtaining more effective treatment options for AD [23]. With this aim, we designed and synthesized a series of heterocyclic compounds by amidation of ethyl piperidine-3-carboxylate (ethyl nipecotate) with various carboxylic acids. Nipecotic acid acts as a GABA reuptake inhibitor and has been used in research for diseases involving GABAergic neurotransmission dysfunction [24]. Carboxylic acids, used as raw materials for synthesis of our compounds, include derivatives of butylate hydroxytoluene (BHT), a widely used antioxidant compound in nutritional and pharmaceutical products. In particular, 3,5-di-tert-butyl-4-hydroxybenzoic acid (BHBA) and ((*E*)-3,5-di-tert-butyl-4-hydroxyphenyl)acrylic acid (BHCA) were used for the synthesis of Compounds **1** and **2** respectively. ((*E*)-3-methoxy-4-hydroxyphenyl)acrylic acid (ferulic acid, for Compound **3**) and ((*E*)-3,5-dimethoxy-4-hydroxyphenyl)acrylic acid (sinapic acid, for Compound **4**) demonstrate neuroprotective effect in cell cultures, mainly due to their antioxidant potency [25,26]. Finally, Compounds **5** and **6** were synthesized from carboxylic acid without presumed antioxidant activity (((*E*)-3,4-dimethoxy-phenyl)acrylic acid and cinnamic acid respectively), but due to the phenylacrylic residue, they are expected to possess anti-inflammatory properties [27].

**Figure 1:**
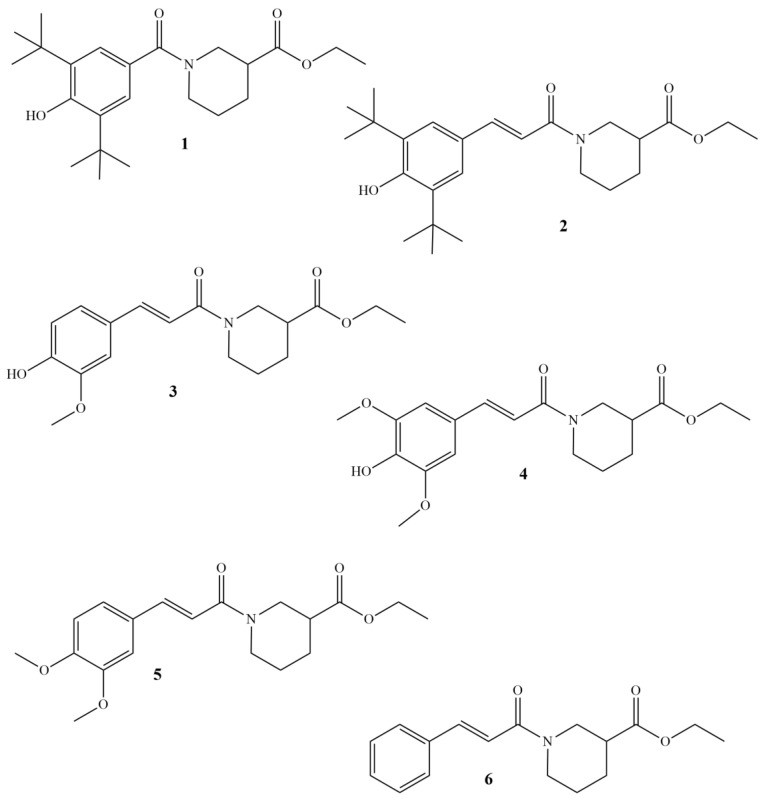
Structures of the synthesized compounds.

All the synthesized compounds (Figure 1) were evaluated for their antioxidant properties through their capacity to inhibit rat hepatic microsomal membrane lipid peroxidation and their reducing activity towards the stable free radical 2,2-diphenyl-1-picrylhydrazyl (DPPH). Furthermore, their activity against fructose-induced protein glycation, was tested. Their inhibitory activity against acetylcholinesterase, soybean lipoxygenase, and their effect on carrageenan-induced rat paw oedema were also evaluated.

## 2. Results

The designed compounds were examined for their inhibitory activity against rat hepatic microsomal membrane lipid peroxidation, induced by Fe^2+^/ascorbic acid and estimated as 2-thiobarbituric acid (TBA) reactive material. The IC_50_ values of compounds after 45 min of incubation are shown in Table 1.

**Figure 2:**
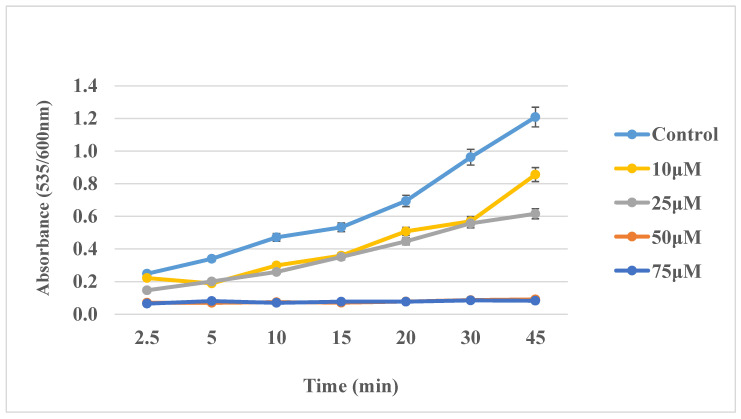
Time course of lipid peroxidation, as affected by Compound **2** (various concentrations).

The effect of Compound **2** (the most active compound) on lipid peroxidation as a function of time is shown in Figure 2.

The antioxidant activity of our compounds was evaluated by their interaction with the stable, N-centred 1,1-diphenyl-2-picrylhydrazyl (DPPH) free radical. The percent interaction between the compounds and DPPH is shown in Table 2.

**Figure 3:**
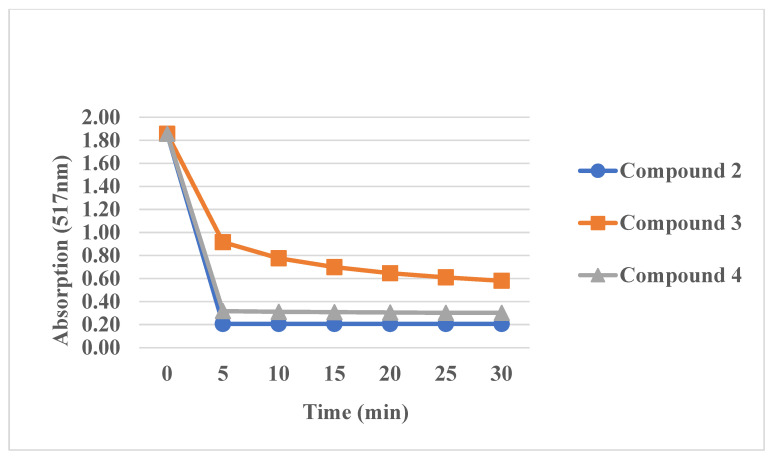
Effect of Compounds **2**–**4**.

Compounds **2**–**4** interacted with DPPH at various concentrations. For Compounds **2** and **4**, the reaction was quite fast, as it was completed during the first 5 min. Compound **3** reacted with DPPH more slowly since the reaction was almost complete after 20 min of incubation (Figure 3).

Furthermore, the antioxidant potential of our compounds was examined by their ability to inhibit oxidative protein glycation, induced by copper cations. The compounds were dissolved in water, but a small amount of dimethylsulfoxide (DMSO) was used in some cases, due to low water solubility. DMSO had no effect on the glycation process. The inhibitory activity against protein glycation of Compounds **1**–**6** is shown in Table 3.

The inhibitory activity of the designed compounds against acetylcholinesterase and soybean lipoxygenase is shown in Table 4. AchE inhibition is expressed as IC_50_ value, whereas LOX inhibition is expressed as % inhibitory activity (concentration 100 μΜ). Physostigmine and nordihydroguairetic acid were used as reference compounds for AchE and LOX inhibition, respectively.

In addition, the anti-inflammatory effect of the compounds was evaluated in vivo by their inhibition against carrageenan-induced rat paw oedema. The effect of the compounds on paw oedema, as well as the respective activity of some NSAIDs used as reference compounds are shown in Table 5. The effect on paw edema was estimated as % edema reduction compared to controls, which were administered only carrageenan. All compounds were administered i.p. at 0.15 mmol/kg of body weight.

Finally, some physicochemical parameters of the compounds are listed in Table 6. All parameters were calculated using Molinspiration.

## 3. Discussion

**Scheme 1:**
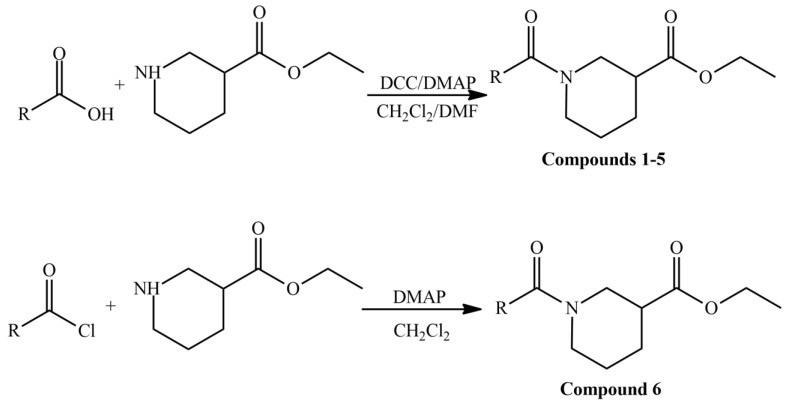
Synthesis of Compounds **1**–**6**.

As shown in Scheme 1, ethyl piperidine-3-carboxylate was amidated with BHBA (for Compound **1**), BHCA (for Compound **2**), ferulic acid (for Compound **3**), sinapic acid (for Compound **4**), and *(E)*-3,4-dimethoxy-phenyl)acrylic acid (for Compound **5**) using N,N-dicyclohexylcarbodiimide (DCC) as a coupling agent and N,N-dimethyl-aminopyridine (DMAP). We have previously reported the synthesis of similar amide derivatives using the present synthetic procedure [28]. The reactions were performed using dichloromethane as a solvent, but a small amount of N-N-dimethylformamide (DMF) was added for Compounds **3**–**5**, because of low solubility of carboxylic acid, used as starting material. The expected products were obtained after purification with flash column chromatography in yields up to 82%. Compound **6** was synthesized by amidation of the commercially available cinnamyl chloride with ethyl nipecotate in dichloromethane. It was obtained in high yield (90%) after purification with flash column chromatography.

As for lipid peroxidation, Compounds **5** and **6** did not demonstrate any antioxidant effect since their structures are not expected to offer them antioxidant capacity. Compound **2** was the most active, exerting similar lipid peroxidation inhibitory activity with trolox, which is a vitamin E analogue with high antioxidant efficacy [29]. Compound **1** had also inhibitory activity against lipid peroxidation, demonstrating half the activity of Compound **2**. Both compounds were expected to possess antioxidant properties, since they are phenolic compounds with high lipophilicity. In previous studies, we have reported BHBA and BHCA derivatives, which could also inhibit lipid peroxidation, with lower or equal activity in some cases compared with the present compounds [28,30]. The differences in the activity may be associated with the relatively high lipophilicity of gabapentin derivatives [28] that leads to low dissolution under the experimental conditions, or the high polar surface area of gamma-aminobutyric acid (GABA) derivatives [30], which may not allow the compounds to effectively approach the microsomal membranes. Comparing Compounds **1** and **2**, the BHCA derivative demonstrated higher antioxidant activity, which can be attributed to the more extended stabilization of the phenolic radical in the phenylacrylic moiety. Compound **3** was not as active as the other compounds, probably because the methoxy substituent has a lower electron donating effect than the di-*tert*-butyl group. Moreover, its lower inhibitory activity may be attributed to the low lipophilicity, which does not allow an effective approach to the lipid environment of microsomal membranes. Finally, the mesomeric effect of the two methoxy substituents in Compound **4** leads to a more stable phenolic radical compared with the monomethoxy substituent in Compound **3**; therefore, the sinapic acid derivative (Compound **4**) was more active against lipid peroxidation than the ferulic derivative (Compound **3**).

In addition, Compounds **2**–**4** could interact with DPPH even at low concentrations (50 μΜ, 1/4 of DPPH concentration). Compound **2** was found to be the most active since it had similar activity with trolox at 200 μM (equal concentration to DPPH). Its high lipophilicity allows it to approach DPPH effectively and interact with it, producing a stable phenolic radical due to the positive inductive effect of the *tert*-butyl substituents. Lipophilicity seems to be a significant property for interaction with DPPH since BHCA derivatives with high lipophilicity have shown to be effective DPPH reducing agents [28,31]. In contrast, Compound **1** produces a less stable phenolic radical after hydrogen abstraction and thus it could not reduce DPPH effectively even at 200 μΜ. Compound **4** also interacted with DPPH even at 50 μΜ, exerting slightly lower activity than Compound **2**. Both compounds could react very rapidly with DPPH and the respective reactions were almost completed during the first five minutes. Taken together with their inhibitory activity against lipid peroxidation, Compounds **2** and **4** were the most potent antioxidant agents among the designed molecules. Compound **3** produces a phenolic radical of lower stability compared with the radicals produced by Compounds **2** and **4**, therefore it exerted lower DPPH reducing efficacy in all the tested concentrations. In addition, it did not react as rapidly with DPPH as Compounds **2** and **4**. Finally, Compounds **5** and **6** do not possess any structural characteristics that would confer on them antioxidant potential, and therefore they did not react with DPPH.

The antioxidant potency of our compounds was also estimated by their inhibitory effect against Cu^2+^-induced oxidative protein glycation. Although it did not demonstrate as high an antioxidant capacity as a lipid peroxidation inhibitor and as a DPPH reducing agent, Compound **3** exerted the highest inhibitory activity against protein glycation, similar to aminoguanidine. The anti-glycation activity can be attributed to the large number of H-bond acceptor substituents, which allow an effective approach between the compound and bovine albumin [32]. Compounds **2** and **4**, despite their higher activity in the other antioxidant experiments, possessed lower inhibitory efficacy against protein glycation. These relatively unaligned results can be attributed to the more rigid structures of Compounds **2** and **4**, which may render them unable to approach bovine albumin effectively. The low water-solubility of Compound **2** is another factor that prevents its effective approach to albumin since the incubation mixture is aqueous. Compound **1** combines a rigid structure with low water solubility and weak antioxidant potency, as evaluated in the other antioxidant experiments. As a result, it did not inhibit protein glycation. Compounds **5** and **6**, lacking antioxidant properties, also exerted no inhibitory activity.

**Figure 4:**
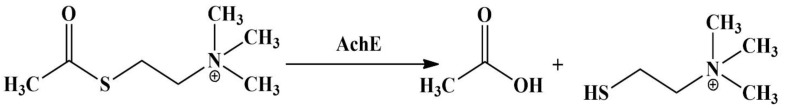
Acetylthiocholine breakdown.

Acetylcholinesterase (AchE) catalyzes acetylcholine cleavage to choline and acetate. Since cholinergic neuron degeneration leads to cognitive impairment, AchE is the pharmacological target of most drugs used clinically against AD [33]. The inhibitory activity of our compounds was estimated by their inhibition of acetylthiocholine breakdown (Figure 4), mediated by AchE contained in a rat brain homogenate.

Compound **4** was the most active against AchE, whereas Compounds **3** and **5** could also inhibit the enzyme effectively. No inhibitory activity was demonstrated when higher concentration of acetylthiocholine was added to the incubation mixture, indicating that our compounds act as competitive AchE inhibitors. The inhibition may be attributed to hydroxy and methoxy substituents on the aromatic ring, which potentially contribute to the binding of the molecule. Furthermore, the piperidine moiety may allow the molecules to bind more effectively to the active site of the enzyme, like donepezil. Sang et al. have also reported AchE inhibitory activity of ferulic derivatives containing a piperidine moiety [34]. Compound **4** contains three aromatic substituents, and thus maybe binds more effectively to the enzyme compared with Compounds **3** and **5**, which have two substituents on the aromatic ring. The lower activity of Compound **1** could result from its larger molecular volume, which does not allow the molecule to approach the active site and bind to it. Compound **2** has an even larger molecular volume, and it exerted no inhibitory activity, possibly due to excessive steric hindrance. Finally, Compound **6** also possessed no activity against AchE. as, it does not contain any aromatic substituents that could allow it to bind effectively to the enzyme.

Lipoxygenases catalyse arachidonic acid metabolism. All isoforms of LOX have a common mechanism of action, which includes arachidonic or linoleic acid peroxidation mediated by molecular oxygen [35]. Lipoxygenase activity is strongly associated with AD pathogenesis, as it seems to increase in older people and is related to both amyloid peptide aggregation and neurofibrillary tangle formation [36,37].

All compounds were moderate soybean LOX inhibitors (up to 33% at 100 μΜ). Compound **2**, which exerted significant antioxidant potential as a lipid peroxidation inhibitor, DPPH reducing agent, and oxidative protein glycation inhibitor, was the most active compound against LOX. Compound **1**, despite the absence of good antioxidant properties, possessed similar activity as a LOX inhibitor to Compound **2**. We can assume that the inhibitory activity is mainly attributed to the binding of the compounds to the enzyme and the prevention of substrate binding and not to the reduction of free radicals produced by LOX. No inhibitory activity was demonstrated when higher concentrations of the substrate (linoleic acid) were used; therefore, we conclude that our compounds act as weak competitive LOX inhibitors. Lipophilicity seems to play a key role for inhibitory activity, as the most active Compounds **1** and **2** have the highest milogP values. Moreover, di-*tert*-butylphenol analogues have been found to exert increased LOX inhibitory activity in previous studies [38]. Compounds **3**–**5**, with lower lipophilicity, possess lower activity than Compounds **1** and **2**. The low activity of nipecotic acid derivatives designed in the present study compared to other amides of antioxidant carboxylic acids with less bulky substituents [39] indicate that large molecular volume is a deterrent factor for inhibitory activity against LOX.

Finally, our compounds were tested for their effect against carrageenan-induced rat paw oedema, a well-known experimental protocol for acute inflammation. Inflammatory response to carrageenan begins with serotonin and histamine release (1.5 h after the injection) and progresses with kinins secretion (about 2.5 h after the administration). The third phase (more than 2.5 h after administration) is characterized by prostaglandin production and pro-inflammatory cytokines release [40]. In our work, paw oedema was evaluated 3.5 h after carrageenan injection. All tested compounds could reduce paw oedema significantly. Compounds **1** and **2** showed extremely strong inhibitory activity, reducing paw oedema by 55% and 61% respectively. Di-*tert*-butylphenol analogues have been found to act as dual LOX/COX2 inhibitors [41], and this fact could explain the high in vivo anti-inflammatory activity of these agents. Compounds **3** and **4** demonstrated significant efficacy, but not so great as Compounds **1** and **2**. All these four compounds were also found to possess antioxidant properties in our experiments, which may contribute to acute inflammation reduction, as we have previously reported [42]. Despite not exerting antioxidant potential, Compounds **5** and **6** could also inhibit rat paw oedema, since not only oxidative stress but various signaling pathways are involved in this process. Finally, taken together the low inhibitory activity of our compounds against LOX with the significant reduction of paw oedema, we can assume that not only LOX, but also many other factors, may be associated with the carrageenan-induced inflammatory reaction All rats administered the test compounds appeared normal after the end of the experimental procedure, both macroscopically and by autopsy. 

## 4. Materials and Methods

### 4.1. General

All commercially available chemicals of the appropriate purity were purchased from Sigma (St. Louis, MO, USA) or Merck (Kenilworth, NJ, USA). The ^1^H-NMR and ^13^C-NMR spectra were recorded using an AGILENT DD2-500 MHz (Santa Clara, CA, USA) spectrometer. Chemical shifts were reported in *δ* (ppm) and signals were given as follows: s, singlet; d, doublet; t, triplet; m, multiplet. Melting points (mp) were determined with a MEL-TEMPII apparatus, Laboratory Devices, Sigma-Aldrich (Milwaukee WI, USA) and were uncorrected. The microanalyses were performed on a Perkin-Elmer 2400 CHN elemental analyzer (Waltham, MA, USA). Thin-layer chromatography (TLC silica gel 60 F254 aluminum sheets, Merck (Kenilworth, NJ, USA) was used to follow the reactions and the spots were visualized under UV light.

### 4.2. Synthesis

General Procedure for the synthesis of Compounds **1**–**5**

The respective carboxylic acid, used as starting material (3 mmol), was dissolved in dry CH_2_Cl_2_. For Compounds **3** and **4** a small quantity of DMF (up to 0.5 mL) was added, due to reduced solubility of the carboxylic acid. Then, ethyl 3-piperidinecarboxylate (3.6 mmol) and N,N-dimethylaminopyridine (DMAP, 3.6 mmol) were added. After 15 min N,N dicyclohexylcarbodiimide (DCC, 3.6 mmol) was added and the mixture was stirred at room temperature overnight. Thereinafter, the mixture was filtered, washed successively with HCl (5%), NaHCO_3_ (5%) and saturated NaCl solution and the organic layer was dried over Na_2_SO_4_. Finally, the solvent was evaporated under reduced pressure and the final compounds were isolated with flash column chromatography using mixtures of ethyl acetate and petroleum ether as eluents [28].

Synthesis of Compound **6**

Cinnamyl chloride (3 mmol) was dissolved in dry CH_2_Cl_2_ and mixed with ethyl 3-piperidinecarboxylate (3.6 mmol) and DMAP (3.6 mmol). The mixture was stirred at ambient temperature overnight, washed successively with HCl (5%), NaHCO_3_ (5%) and saturated NaCl solution, and the organic layer was dried over Na_2_SO_4_. Finally, the solvent was removed under reduced pressure and the final compound was isolated with flash column chromatography using a mixture of petroleum ether and ethyl acetate as eluents.

Ethyl 1-(3,5-di-tert-butyl-4-hydroxybenzoyl)piperidine-3-carboxylate (**1**): Flash Column Chromatography (ethyl acetate/petroleum ether 1/2). Yellow powder, yield 73%, mp 47–49 °C. ^1^H-NMR (CDCl_3_). δ(ppm): 7.72 (s, 2H, aromatic), 5.41 (s, 1H, -OH), 4.13 (q J: 7.1 Hz, 2H, -OC**H_2_**-CH_3_), 3.07–3.15 (m, 1H, 6-piperidine), 2.92–2.99 (m, 1H, 6-piperidine), 2.56 (t J: 10.8, 1H, 3-piperidine), 2.12 (dd J: 16.3 Hz, 12.7 Hz, 2H, 2-piperidine), 1.49–1.81 (m, 4H, 4/5-piperidine), 1.43 (s, 18H, -C(CH_3_)_3_), 1.24 (t J: 7.1 Hz, 3H, -OCH_2_-C**H_3_**). ^13^C-NMR (CDCl_3_). δ(ppm): 173.07 (1C, -**C**OOCH_2_CH_3_), 171.63 (1C, -CO-NH-), 155.37 (1C, 4-aromatic), 136.67 (2C, 3/5-aromatic), 126.29 (1C, 1-aromatic), 124.53 (2C, 2/6-aromatic), 60.64 (1C, -O**C**H_2_CH_3_), 45.67 (2C, 2/6-piperidine), 41.82 (1C, 3-piperidine), 34.35 (2C, -**C**(CH_3_)_3_), 30.11 (6C, -C(**C**H_3_)_3_), 27.57 (1C, 4-piperidine), 24.66 (1C, 5-piperidine), 14.20 (1C, -OCH_2_**C**H_3_). Anal. Calculated for C_23_H_35_NO_4_: C, 70.92; H, 9.06; N, 3.60%. Found C, 70.72; H, 9.08; N, 3.71%.

(*E*)-Ethyl 1-(3-(3,5-di-tert-butyl-4-hydroxyphenyl)acryloyl)piperidine-3-carboxylate (**2**): Flash Column Chromatography (ethyl acetate/petroleum ether 1/3). Yellow powder, yield 82%, mp 158–161 °C. ^1^H-NMR (CDCl_3_). δ(ppm): 7.61 (d J: 15.3 Hz, 1H, Ph-**C**H=CH), 7.34 (s, 2H, aromatic), 6.73 (d J: 15.3 Hz, 1H, Ph-CH=**C**H) 5.44 (s, 1H, -OH), 4.15 (q J: 7.1 Hz, 2H, -O**C**H_2_CH_3_), 3.94–4.05 (m, 1H, 6-piperidine), 3.43–3.60 (m, 1H, 6-piperidine), 2.88–3.17 (m, 2H, 2-piperidine), 2.52 (t, J: 10.0 Hz, 1H, 3-piperidine), 2.05–2.10 (m, 1H, 5-piperidine), 1.72–1.84 (m, 2H, 4-piperidine), 1.52–1.58 (m, 1H, 5-piperidine), 1.45 (s, 18H, -C(CH_3_)_3_), 1.26 (t J: 7.1 Hz, 3H, -OCH_2_C**H_3_**). ^13^C-NMR (CDCl_3_). δ(ppm): 173.67 (1C, -**C**OOCH_2_CH_3_), 166.12 (1C, -CO-NH-), 155.46 (1C, 4-aromatic), 143.24 (1C, Ph-**C**H=CH-), 136.20 (2C, 3/5-aromatic), 126.62 (1C, 1-aromatic), 124.96 (2C, 2/6-aromatic), 113.89 (1C, Ph-CH=**C**H-), 60.67 (1C, -O**C**H_2_CH_3_), 45.96 (2C, 2/6-piperidine), 41.96 (1C, 3-piperidine), 34.30 (2C, -**C**(CH_3_)_3_), 30.17 (6C, -C(**C**H_3_)_3_), 27.43 (1C, 4-piperidine), 24.68 (1C, 5-piperidine), 14.17 (1C, -OCH_2_**C**H_3_). Anal. Calculated for C_25_H_37_NO_4_: C, 72.26; H, 8.97; N, 3.37. Found C, 72.54; H, 9.01; N, 3.09%.

(*E*)-Ethyl 1-(3-(4-hydroxy-3-methoxyphenyl)acryloyl)piperidine-3-carboxylate (**3**): Flash Column Chromatography (ethyl acetate/petroleum ether 1/1). Yellow oily liquid, yield 64%. ^1^H-NMR (CDCl_3_). δ(ppm): 7.59 (d J: 15.3 Hz, 1H, Ph-**C**H=CH), 7.09 (d J: 8.1 Hz, 1H, 6-aromatic), 6.99 (s, 1H, 2-aromatic), 6.90 (d J: 8.1 Hz, 1H, 5-aromatic), 6.77 (d J: 15.3 Hz, 1H, Ph-CH=**C**H), 5.41 (s, 1H, -OH), 4.15 (q J: 7.1 Hz, 2H, -O**C**H_2_CH_3_), 3.92 (s, 3H, Ph-OC**H_3_**), 3.10–3.19 (m, 1H, 6-piperidine), 2.52 (t, J: 9.7 Hz, 1H, 3-piperidine), 2.03–2.17 (m, 1H, 6-piperidine), 1.48–1.97 (m, 6H, 2/4/5-piperidine), 1.26 (t J: 7.1 Hz, 3H, -OCH_2_C**H_3_**). ^13^C-NMR (CDCl_3_). δ(ppm): 172.89 (1C, -**C**OOCH_2_CH_3_), 166.85 (-CO-NH-), 147.67 (1C, 4-aromatic), 142.27 (1C, 3-aromatic), 137.64 (1C, Ph-**C**H=CH), 127.84 (1C, 1-aromatic), 121.90 (1C, 6-aromatic), 114.70 (1C, Ph-CH=**C**H), 114.66 (1C, 5-aromatic), 109.86 (1C, 2-aromatic), 60.79 (1C, -O**C**H_2_CH_3_), 55.99 (1C, Ph-O**C**H_3_), 45.11 (2C, 2/6-piperidine), 43.86 (1C, 3-piperidine), 27.43 (1C, 4-piperidine), 25.56 (1C, 5-piperidine), 14.17 (1C, -OCH_2_**C**H_3_). Anal. Calculated for C_18_H_23_NO_5_: C, 64.85; H, 6.95; N, 4.20%. Found C, 64.99; H, 7.18; N, 3.82%.

(*E*)-Ethyl 1-(3-(4-hydroxy-3,5-dimethoxyphenyl)acryloyl)piperidine-3-carboxylate (**4**): Flash Column Chromatography (ethyl acetate/petroleum ether 1/1). Yellow powder, yield 65%, mp 59–61 °C. ^1^H-NMR (CDCl_3_). δ(ppm): 7.57 (d J:15.3 Hz, 1H, Ph-C**H**=CH), 6.76 (s, 2H, aromatic), 6.74 4 (d J:15.3 Hz, 1H, Ph-CH=C**H**), 4.15 (q J: 7.1 Hz, 2H, -OC**H_2_**CH_3_), 3.92 (s, 6H, Ph-OC**H_3_**), 3.11–3.20 (m, 1H, 6-piperidine), 2.53 (t J: 9.7 Hz, 1H, 3-piperidine), 1.53–2.09 (m, 7H, 2/4/5/6-piperidine), 1.26 (t J: 7.1 Hz, 3H, -OCH_2_C**H_3_**). ^13^C-NMR (CDCl_3_). δ(ppm): 173.11 (1C, -**C**OOCH_2_CH_3_), 166.73 (1C, -CO-NH-), 147.16 (2C, 3/5-aromatic), 143.25 (1C, 4-aromatic), 136.54 (1C, Ph-**C**H=CH), 126.78 (1C, 1-aromatic), 114.93 (1C, Ph-CH=**C**H), 104.83 (2C, 2/6-aromatic), 60.76 (1C, -O**C**H_2_CH_3_), 56.38 (2C, Ph-O**C**H_3_), 41.56 (2C, 2/6-piperidine), 36.72 (1C, 3-piperidine), 27.43 (2C, 4/5-piperidine), 14.18 (1C, -OCH_2_**C**H_3_). Anal. Calculated for C_19_H_25_NO_6_: C, 62.80; H, 6.93; N, 3.85%. Found C, 63.12; H, 7.26; N, 3.67%.

(*E*)-Ethyl 1-(3-(3,4-dimethoxyphenyl)acryloyl)piperidine-3-carboxylate (**5**): Flash Column Chromatography (ethyl acetate/petroleum ether 1/1). Yellow oily liquid, yield 78%. ^1^H-NMR (CDCl_3_). δ(ppm): 7.61 (d J: 15.3 Hz, 1H, Ph-C**H**=CH), 7.11 (d J: 8.3 Hz, 1H, 6-aromatic), 7.03 (s, 1H, 2-aromatic), 6.86 (d J: 8.3 Hz, 1H, 5-aromatic), 6.80 (d J: 15.3 Hz, 1H, Ph-CH=C**H**), 4.15 (q J: 7.1 Hz, 2H, -OC**H_2_**CH_3_), 3.97-4.08 (m 1H, 6-piperidine), 3.92 (s, 3H, Ph-OC**H_3_**), 3.90 (s, 3H, Ph-OC**H_3_**), 3.45–3.60 (m, 1H, 6-piperidine), 3.12–3.19 (m, 1H, 2-piperidine), 2.52 (t J: 9.8 Hz, 1H, 3-piperdidine), 2.05–2.11 (m, 1H, 2-piperidine), 1.52–1.89 (m, 4H, 4/5-piperidine), 1.26 (t J: 7.1Hz, 3H, -OCH_2_C**H_3_**). ^13^C-NMR (CDCl_3_). δ(ppm): 173.66 (1C, -**C**OOCH_2_CH_3_), 166.78 (1C, -CO-NH-), 150.47 (1C, 4-aromatic), 149.07 (1C, 3-aromatic), 142.79 (1C, Ph-**C**H=CH), 128.28 (1C, 1-aromatic), 121.77 (1C, 6-aromatic), 114.97 (1C, Ph-CH=**C**H), 111.03 (1C, 5-aromatic), 109.87 (1C, 2-aromatic), 60.74 (1C, -O**C**H_2_CH_3_), 55.94 (2C, Ph-O**C**H_3_), 52.83 (1C, 2-piperidine), 44.12 (1C, 6-piperidine), 43.09 (1C, 3-piperidine), 27.44 (1C, 4-piperidine), 24.02 (1C, 5-piperidine), 14.19 (1C, -OCH_2_**C**H_3_). Anal. Calculated for C_19_H_25_NO_5_: C, 65.59; H, 7.25; N, 4.03%. Found C, 65.38; H, 7.00; N, 4.13%.

Ethyl 1-cinnamoylpiperidine-3-carboxylate (**6**): Flash Column Chromatography (ethyl acetate/petroleum ether 1/3). Colorless oily liquid, yield 85%. ^1^H-NMR (CDCl_3_). δ(ppm): 7.68 (d J: 15.4 Hz, 1H, Ph-C**H**=CH), 7.51–7.57 (m, 2H, aromatic), 7.33–7.41 (m, 2H, aromatic), 6.94 (d J: 15.4 Hz, 1H, Ph-CH=**C**H), 4.17 (q J: 7.1 Hz, 2H, -OC**H_2_**CH_3_), 4.03–4.10 (m, 1H, 6-piperidine), 3.18–3.38 (m, 2H, 2/6-piperidine), 2.52 (t J: 9.8 Hz, 1H, 3-piperdidine), 2.06–2.14 (m, 1H, 2-piperidine), 1.52–1.86 (m, 4H, 4/5-piperidine), 1.27 (t J: 7.1 Hz, 3H, -OCH_2_C**H_3_**). ^13^C-NMR (CDCl_3_). δ(ppm): 172.99 (1C, -**C**OOCH_2_CH_3_), 166.68 (1C, -CO-NH-), 142.23 (1C, Ph-**C**H=CH), 135.27 (1C, 1-aromatic), 129.60 (1C, 4-aromatic), 128.76 (2C, 3/5-aromatic), 127.77 (2C, 2/6-aromatic), 117.19 (1C, Ph-CH=**C**H), 60.79 (1C, -O**C**H_2_CH_3_), 52.59 (1C, 2-piperidine), 46.40 (1C, 6-piperidine), 41.60 (1C, 3-piperidine), 27.36 (1C, 4-piperidine), 24.54 (1C, 5-piperidine), 14.19 (1C, -OCH_2_**C**H_3_). Anal. Calculated for C_17_H_23_NO_3_: C, 71.06; H, 7.37; N, 4.87%. Found C, 71.33; H, 7.76; N, 4.75%.

### 4.3. Biological Evaluation

κ-Carrageenan and lipoxygenase type I-B from soybean were purchased from Sigma (St. Louis, MO, USA). For the in vivo experiments, Wistar rats (180–220 g) were kept in the Centre of the School of Veterinary Medicine (EL54 BIO42), Aristotle University of Thessaloniki, which is registered by the official state veterinary authorities (presidential degree 56/2013, in harmonization with the European Directive 2010/63/EEC). The experimental protocols were approved by the Animal Ethics Committee of the Prefecture of Central Macedonia (no. 270079/2500).

#### In Vitro Lipid Peroxidation Inhibition

The peroxidation of rat liver microsomal fraction, inactivated after heating (90 °C, 90 s), was induced by ascorbic acid (0.2 mM) and FeSO_4_ (10 µM). The test compounds, in dimethylsulfoxide, were added at concentrations of 1 µM to 1 mM. Aliquots were taken from the incubation mixture (37 °C) for 45 min. Lipid peroxidation was assessed spectrophotometrically (535/600 nm) as 2-thiobarbituric acid reactive material. All compounds and solvents were found not to interfere with the assay [30].

*In vitro interaction with the Stable Free Radical 1,1-Diphenyl-2-Picrylhydrazyl (DPPH)*

Compounds (in absolute ethanol, final concentrations 50–200 µM) were added to an ethanolic solution of DPPH (final concentration 200 µM) at ambient temperature (22 ± 2 °C). Absorbance (517 nm) was recorded every 5 min for 30 min [39].

*In vitro Protein Glycation Inhibition*

Incubations of bovine serum albumin (BSA 4 mg/mL) were carried out with fructose (250 mM) in 120 mM phosphate buffer (pH 7.4), in the presence of Cu^2+^ (10 μΜ) and NaN_3_ (0.015%) at 37 °C for 72 h. The incubations were carried out at least in triplicate parallel tubes. Glycation-modified protein purification was based on protein precipitation and washings with trichloroacetic acid (TCA). Fluorescence measurements were made in 60 mM phosphate buffer (pH 7.4) at an excitation wavelength of 340 nm, with an emission wavelength of 410nm, and expressed relatively to the standard quinine sulphate solution (1 μg/mL). Incubations carried out under the same conditions but in the absence of fructose were used as controls [28]. All compounds were found to be stable under the conditions of the experiment.

*In vitro Evaluation of Acetylcholinesterase Activity*

Brains from untreated rats were homogenized (20 mg/mL) in phosphate buffer (0.1 M, pH 8). Acetylcholinesterase activity was assessed using brain homogenate, acetylthiocholine (0.5 mM) as a substrate, in the presence of the tested compounds (dissolved in 60% ethanol) and evaluating the reaction product of the liberated thiocholine with DTNB at 412 nm. The used solvent system was tested and found not to interfere with the assay [28].

*In vitro Evaluation of Lipoxygenase Activity*

The reaction mixture contained the examined compounds (in absolute ethanol), soybean lipoxygenase (in saline, 250 u/mL) and sodium linoleate (100 µM), in Tris–HCl buffer, pH 9.0. The reaction was monitored for 7 min at 28 °C, recording the absorbance at 234 nm. Nordihydroguaiaretic acid (NDGA) was used as a reference [43].

*In vivo Evaluation of Anti-Inflammatory Activity*

The tested compounds (in water with a few drops of Tween 80) were administered i.p. (0.15 mmol/kg) to rats, just after the i.d. injection of 0.1 mL of an aqueous carrageenan solution (1% *w*/*v*) in the hind paw of rats. The produced oedema, after 3.5 h, was estimated as paw weight increase [44].

## 5. Conclusions and Future Perspectives

There is much evidence in the literature that oxidative stress and inflammatory responses are both key factors for the pathogenesis of neurodegeneration [10,11,12,13,14,15,16,17,18,19]. Alzheimer’s disease is the most common neurodegenerative disorder, characterized by memory loss and cognitive impairment. It is a multi-factorial disease, and its pathophysiology has not been fully elucidated. Therefore, no effective pharmacological treatment has been obtained yet. Multi-targeting compounds could prove to be useful therapeutic agents against AD, since they affect more than one biochemical pathways related to its development and progression.

In our work we designed and synthesized a series of ethyl nipecotate amides, which were tested for their antioxidant properties, their anti-inflammatory potential, and their ability to inhibit acetylcholinesterase. Compound **2** (the BHCA derivative) demonstrated significant antioxidant potential, mainly as a lipid peroxidation inhibitor and as a DPPH reducing agent, due to its tert-butyl-groups, which are lipophilic substituents and stabilize the produced phenolic radical. Moreover, it was the most active compound against lipoxygenase, and could reduce carrageenan-induced rat paw oedema by 61%. Compound **1**, despite the tert-butyl substituents, did not possess as high an antioxidant capacity, but it had similar anti-inflammatory properties to Compound **2** and could moderately inhibit acetylcholinesterase. Methoxy substituted phenolic Compounds **3** and **4** demonstrated lower antioxidant and anti-inflammatory capacity compared to Compound **2**, but they were shown to be significantly more active as acetylcholinesterase inhibitors. Compound **5**, did not possess any antioxidant activity, as expected, but it could inhibit acetylcholinesterase and reduce rat paw oedema. Finally, compound 6 could only reduce rat paw oedema, as it had no antioxidant potential and could not inhibit acetylcholinesterase.

The combination of antioxidant and anti-inflammatory potency with acetylcholinesterase inhibitory activity, could prove useful for further development of anti-Alzheimer therapeutic agents. We hope that our compounds can contribute towards this direction, and effective therapeutic options may be obtained after optimization.

## Figures and Tables

**Table 1:** IC_50_ values of Compounds **1**–**6** and trolox against lipid peroxidation.

Compound	IC_50_ (μΜ)
**1**	39
**2**	20
**3**	160
**4**	47
**5**	-
**6**	-
**Trolox**	25

After 45 min incubation. Trolox: 6-hydroxy-2,5,7,8-tetramethylchroman-2-carboxylic acid. All determinations were performed at least in triplicate and the standard deviation was always within ±10% of the mean value, -: inactive. SEM values were between 0.004–0.010.

**Table 2:** Interaction of Compounds **1**–**6** and trolox, at various concentrations, with DPPH (200 μΜ).

Compound	% Interaction with DPPH		
	200 μΜ	100 μΜ	50 μΜ
**1**	10	-	-
**2**	89	50	33
**3**	69	37	27
**4**	84	47	32
**5**	-	-	-
**6**	-	-	-
**Trolox**	92	90	38

After 30 min incubation. Trolox: 6-hydroxy-2,5,7,8-tetramethylchroman-2-carboxylic acid. All determinations were performed at least in triplicate and the standard deviation was always within ±10% of the mean value. -: inactive. SEM values were between 0.003–0.008.

**Table 3:** Percentage inhibition of Compounds **1**–**6** and aminoguanidine (concentration 1 mM) against protein glycation.

Compound	% Inhibition
**1**	-
**2**	31
**3**	57
**4**	41
**5**	-
**6**	-
**Aminoguandine**	56

After 72 h incubation. All determinations were performed at least in triplicate and the standard deviation was always within ±10% of the mean value. -: inactive. SEM values were between 0.03–0.14.

**Table 4:** Inhibitory activity of Compounds **1**–**6** and reference compounds against acetylcholinesterase (IC_50)_ and LOX (% inhibition at 100 μΜ).

Compound	IC_50_ (μΜ) (AchE)	% Inhibition (LOX)
**1**	192	31
**2**	-	33
**3**	86	24
**4**	47	20
**5**	74	24
**6**	-	15
**Reference Compound**	0.15	94

After 7 min incubation for both experiments. All determinations were performed at least in triplicate and the standard deviation was always within ±10% of the mean value. -: inactive. SEM values were between 0.04–0.09.

**Table 5:** Effect of Compounds **1**–**6**, ibuprofen, and naproxen, on carrageenan-induced rat paw oedema.

Compound	% Oedema Reduction
**1**	55 **
**2**	61 ***
**3**	47 **
**4**	49 ***
**5**	37 ***
**6**	35 ***
**Ibuprofen**	36 *
**Naproxen**	11 *

Significant difference from control: * *p* < 0.01, ** *p* < 0.005, *** *p* < 0.001 (Student’s *t* test). SEM values were between 0.69–1.57.

**Table 6:** Physicochemical parameters of Compounds **1**–**6**, as calculated via Molinspiration.

Compound	Molecular Weight	Molecular Volume	milogP	TPSA	H-Bond Donors	H-Bond Acceptors	Rotatable Bonds	Violations (Lipinski)
**1**	389.54	390.73	4.96	66.84	1	5	6	0
**2**	415.57	418.14	6.08	66.84	1	5	7	1
**3**	333.38	311.31	2.37	76.08	1	6	6	0
**4**	363.41	336.86	2.38	85.31	1	7	7	0
**5**	347.41	328.84	2.67	65.08	0	6	7	0
**6**	287.36	277.75	3.03	46.61	0	4	5	0

## Data Availability

The data presented in this study are available on request from the corresponding author.
